# Physiological fibrin hydrogel modulates immune cells and molecules and accelerates mouse skin wound healing

**DOI:** 10.3389/fimmu.2023.1170153

**Published:** 2023-04-24

**Authors:** Rafaela Vaz Sousa Pereira, Mostafa EzEldeen, Estefania Ugarte-Berzal, Erik Martens, Bert Malengier-Devlies, Jennifer Vandooren, Jan Jeroen Vranckx, Patrick Matthys, Ghislain Opdenakker

**Affiliations:** ^1^ Laboratory of Immunobiology, Rega Institute for Medical Research/KU Leuven, Department of Microbiology, Immunology and Transplantation, Leuven, Belgium; ^2^ OMFS IMPATH Research Group, University Hospitals Leuven/KU Leuven, Department of Imaging and Pathology, Leuven, Belgium; ^3^ Pediatric Dentistry and Special Dental Care, University Hospitals Leuven/KU Leuven, Department of Oral Health Sciences, Leuven, Belgium; ^4^ Department of Development and Regeneration, University Hospitals Leuven/KU Leuven, Leuven, Belgium; ^5^ Department of Plastic and Reconstructive Surgery, University Hospitals Leuven/KU Leuven, Leuven, Belgium

**Keywords:** wound healing, fibrin, leukocytes, macrophages, inflammation, hydrogel, cytokines

## Abstract

**Introduction:**

Wound healing is a complex process to restore homeostasis after injury and insufficient skin wound healing is a considerable problem in medicine. Whereas many attempts of regenerative medicine have been made for wound healing with growth factors and cell therapies, simple pharmacological and immunological studies are lagging behind. We investigated how fibrin hydrogels modulate immune cells and molecules in skin wound healing in mice.

**Methods:**

Physiological fibrin hydrogels (3.5 mg/mL fibrinogen) were generated, biophysically analyzed for stiffness and protein contents and were structurally studied by scanning electron microscopy. Physiological fibrin hydrogels were applied to full thickness skin wounds and, after 3 days, cells and molecules in wound tissues were analyzed. Leukocytes, endothelial cells, fibroblasts and keratinocytes were explored with the use of Flow Cytometry, whereas cytokines and matrix metalloproteinases were analyzed with the use of qPCR, ELISAs and zymography. Skin wound healing was analyzed microscopically at day 3, macroscopically followed daily during repair in mice and compared with commercially available fibrin sealant Tisseel.

**Results:**

Exogenous fibrin at physiological concentrations decreased neutrophil and increased non-classical Ly6Clow monocyte and resolutive macrophage (CD206^+^ and CX3CR1^+^) populations, at day 3 after injury. Fibrin hydrogel reduced the expression of pro-inflammatory cytokines and increased IL-10 levels. In line with these findings, gelatinase B/MMP-9 was decreased, whereas gelatinase A/MMP-2 levels remained unaltered. Frequencies of dermal endothelial cells, fibroblasts and keratinocytes were increased and keratinocyte migration was enhanced by fibrin hydrogel. Importantly, physiological fibrin accelerated the healing of skin wounds in contrast to the highly concentrated fibrin sealant Tisseel, which delayed wound repair and possessed a higher fiber density.

**Conclusion:**

Collectively, we show that adding a tailored fibrin hydrogel scaffold to a wound bed positively influences the healing process, modulating leukocyte populations and inflammatory responses towards a faster wound repair.

## 1 Introduction

Wound healing is a dynamic and complex process consisting of molecular and cellular components, highly coordinated to restore homeostasis after tissue injury (1). This repair process occurs through consecutive and partially overlapping phases: hemostasis, inflammation, granulation/proliferation, and tissue remodeling. Interruptions and/or dysregulations in one of these phases may impair the healing process and lead to the development of non-healing wounds. Non-healing wound treatments represent a huge burden to health care systems and individual patients, therefore strategies to accelerate the healing of wounds are needed (2, 3).

Following injury, the hemostasis process is the first critical step to protect the host from blood loss. Soluble fibrinogen is cleaved by thrombin into fibrin monomers, which are sequentially cross-linked by Factor XIII (4). This cascade forms the fibrin clot, a provisory extracellular matrix consisting of a network of insoluble fibrin fibers formed within the injury site. The fibrin clot not only controls the bleeding but also acts as a scaffold for cells to migrate into the wound and as a reservoir for growth factors. Moreover, fibrin triggers a wide spectrum of signaling responses by fibrin receptor interactions with different cell types in the wound environment, thereby modulating processes such as migration, proliferation, angiogenesis and re-epithelialization (5). Platelet activation and aggregation are also important effects happening by the fibrin clot. Activated platelets release growth factors, such as platelet-derived growth factor (PDGF) and transforming growth factor-beta (TGF-β), which stimulate the repair and regeneration of damage tissues (6, 7).

After hemostasis, an inflammatory phase follows. This phase is characterized by an early influx of neutrophils at the injury site, followed by other leukocytes, such as monocytes/macrophages and lymphocytes. Macrophages are key players to promote tissue repair and they are mainly derived from recruited circulating monocytes. Macrophages first show a pro-inflammatory phenotype, secrete a variety of inflammatory mediators and promote clearance of debris and pathogens. At later stages of the inflammatory phase, their profile is switched to a more resolutive phenotype with the secretion of anti-inflammatory and angiogenic factors for progression to tissue repair (8). The plasticity of macrophages allows these cells to promote both tissue destructive and reparative functions, and the balance between these functions is crucial to restore tissue integrity (9). It is known that tissue environments actively regulate the polarization of macrophages through soluble and anchored factors within the local environment. Cytokines and chemokines are such prototypic soluble mediators, whereas extracellular matrix and cell adhesion molecules represent anchored entities (10, 11). The transition from inflammation to the granulation/proliferation phase is a critical step for an effective wound healing process (12). In the latter phase, the participation of endothelial cells, fibroblasts and keratinocytes is crucial. During this phase, angiogenesis is necessary for the formation of new blood vessels, while fibroblasts secrete the new extracellular matrix (13). At the same time, migration of keratinocytes enables wound re-epithelialization.

The use of biomaterials, both natural and synthetic, to promote tissue repair and regeneration has increased considerably in the last few years. The interaction of biomaterials with key cellular components of wound healing, such as endothelial cells, fibroblasts and keratinocytes, to promote cell migration and proliferation is an important point to consider when aiming for the use of biomaterials for tissue repair. Additionally, growing interest also exists in understanding the capacity of microenvironmental cues presented by these biomaterials in modulating macrophages phenotypes (14, 15). In this context, the implication of fibrin in wound healing and its interaction with different cell types, including leukocytes, endothelial cells, fibroblasts and keratinocytes, places this natural polymeric protein as a therapeutic biomaterial candidate to modulate the wound environment (5, 16–18). In fact, fibrin sealants are commercially available preparations, which have already been used for hemostasis purposes in surgical procedures for a long time. These commercial preparations consist of high concentrations of fibrinogen and thrombin components and their use on their own for promoting cutaneous wound repair has yielded inconsistent results (19). In addition, several fibrin preparations have been studied in more complex regenerative medicine approaches, i.e. with the combination of cells or therapeutic biomolecules (16, 20). These studies were unsuccessful in defining the modulatory effect of fibrin alone.

To gain insights into the direct role of fibrin itself in modulating endogenous cells *in vivo*, we prepared a nearly physiological fibrin hydrogel scaffold and investigated its effects in a mouse model of skin wound healing. We evaluated fibrin effects on leukocytes and immune molecules as well as on endothelial cells, fibroblasts and keratinocytes in the wound environment. Moreover, we compared biophysical structures and the outcome of the fibrin preparations from our study with the commercially available fibrin sealant.

## 2 Materials and methods

### 2.1 Hydrogel composition and preparation

Fibrin hydrogels with specific biophysical properties were prepared as described previously (21). Briefly, plasminogen-depleted human fibrinogen (Enzyme Research Laboratories, USA) was dissolved in fibrinogen buffer (20 mM HEPES and 150 mM NaCl). Thrombin (Sigma, USA) and factor XIII (Fibrogammin, CSL Behring, Germany) were prepared in thrombin buffer (20 mM HEPES, 150 mM NaCl, 40 mM CaCl_2_ and 0.1% BSA) and kept in a water bath at 37°C for 30 min to activate factor XIII to factor XIIIa. Fibrinogen and thrombin components were mixed to prepare hydrogels with 3.5 mg/ml fibrinogen, 0.1 U/ml thrombin and 0.1 U/ml factor XIII. As a quality control of the used fibrinogen/fibrin materials we investigated the presence of fibronectin in the used fibrinogen preparation and measured fibrin gel stiffness with increasing concentrations of fibrinogen. With the use of total protein staining with Coomassie Brilliant Blue and the comparison with a standard preparation of recombinant human fibronectin (Corning), we determined that the used fibrinogen preparation contained low, but detectable levels of fibronectin. As expected, the fibronectin contaminant migrated at approximately 250 kDa, whereas the alpha-, beta- and gamma-chains of the used human fibrinogen migrated between 70 kDa and 40 kDa (**Supplementary Figure 1A**).

To measure the elastic modulus as a parameter of the stiffness of the hydrogels, we used various concentrations of fibrinogen and determined the stiffness of hydrogels exactly as previously described (21) (**Supplementary Figure 1B**).

The fibrin sealant Tisseel S/D (Baxter, USA) was used in wound healing experiments as a commercially available comparison with our fibrin hydrogel preparations. Because Tisseel has an extremely high fibrinogen concentration, its elastic modulus is about 100 kPa (22).

### 2.2 Wound induction

Full-thickness excisional wounds were induced on the back of C57BL/6J mice. All procedures were conducted in accordance with the regulations of the European Union (directive 2010/63/EU) and were approved by the local Animal Ethics Committee of KU Leuven (License numbers P270/2015, P128/2019). Briefly, mice were anesthetized with ketamine (100 mg/kg) and xylazine (10 mg/kg) and the hair was removed using a shaver and hair removal cream. The skin was lifted at the mid-dorsal line, folded and punched through with a 4mm disposable sterile biopsy punch (Robbins Instruments, USA), creating one wound on each side of the dorsal midline. The procedure was repeated one more time, generating 4 wounds per animal. Fibrin hydrogel or Tisseel was applied locally in a volume of 25 μl per wound while the animal was still anesthetized and kept under an infrared heating lamp. The control mice were not treated. All wounds were left to dry before the mice were returned to their cages. The wounds were not covered with dressing and single mice were housed in individual clean cages to prevent confounding effects caused by co-housed animals. The prevention of infection was by disinfection of the skin, prior to punching, and conventional sterile surgical handling. After wound treatments, all animals were kept in a conventional (non-specific pathogen-free) animal house and no antiseptics were used. The experiments were done with groups of 5 mice and repeated once with an additional 5 mice per group. For statistical analysis all data points were included.

### 2.3 Flow cytometry

Following humane euthanasia, wounds were harvested with the use of a 6 mm punch biopsy, having a larger diameter than the initial wound puncher and we collected both dermis and epidermis layers. To have sufficient cellular materials, pools of 2 wounds from the same mouse were chopped and digested with Liberase TL enzyme cocktail [0.35 mg/mL Liberase TL (Roche, Germany), 3 mg/mL Collagenase D (Roche, Germany) and 0.1 mg/mL DNase I (Roche, Germany)] for 2 hours, at 37°C. After incubation, the samples were filtered through a 70-µm strainer to remove undigested debris and to yield single-cell suspensions. Approximately 1x10^6^ cells were incubated with the Fc-receptor-blocking antibodies anti-CD16/anti-CD32 (BD Biosciences Pharmingen, USA) and with a Zombie Aqua™ viability dye (BioLegend, USA) for 15 min. After washing with FACS buffer (0.5% BSA; 2-mM EDTA in PBS), surface receptors were stained with conjugated antibodies for 30 min (**Supplementary Table 1**). Next, cells were washed and fixed with 0.37% formaldehyde in PBS. Cells were analyzed on a Flow Cytometer BD LSR Fortessa X20 with DIVA software. Results were further analyzed with the FlowJo software (BD). Gating strategies are shown in **Supplementary Figures 2, 3**.

### 2.4 Measurement of mRNA expression by real-time qPCR

RNA was isolated from wound tissue using RNeasy Mini Kit according to the manufacturer’s instructions (Qiagen, Germany). Precellys 24 tissue homogenizer and bulk beads were used to homogenize the tissue (Bertin Instruments, Germany). RNA quality and quantity were verified and cDNA was synthesized using the High Capacity cDNA Reverse Transcription Kit (Applied Biosystems, USA). Relative changes in gene expression were evaluated by qPCR using the TaqMan Fast Universal PCR master mix (Applied Biosystems, USA), amplified, and analyzed on the 7500 Fast Real-time PCR system. The primers used are listed in **Supplementary Table 2**. The relative mRNA expression was determined with the 2^−ΔΔCt^ method, in which 18S RNA was used as a housekeeping gene. Fold changes were then calculated and compared to the mean of the Control group.

### 2.5 Analysis of cytokines in wound tissue

Wound tissues were homogenized in Lysis Buffer compatible with ELISA (50mM Tris, 150mM NaCl and 1% Triton X-100) and supplemented with Protease Inhibitor Cocktail (Thermo Fisher Scientific, USA). Precellys 24 tissue homogenizer and bulk beads were used to homogenize the tissue (Bertin Instruments, Germany). The samples were centrifuged at 10000 G for 5 min at 4°C to harvest the soluble fraction. IL-6, TNF-α, IL-1β, IL-10 and TGF-β were then measured by enzyme-linked immunosorbent assay (ELISA) (R&D systems, UK).

### 2.6 Gelatin zymography analysis

Gelatinases from wound homogenates were pre-purified and analyzed through gelatin zymography as previously described (23). Briefly, 30 μg protein samples from wound homogenates were incubated with gelatin-Sepharose beads (GE Healthcare) on mini-spin columns, which enabled the binding of gelatinases to the gelatin-coated beads. Following the removal of non-bound proteins by subsequent washes, the proteases were directly eluted from the beads into 30 μl of nonreducing and SDS-containing loading buffer. Eluted samples (5 μl) were then run in gels copolymerized with gelatin. A standard internal preparation with recombinant proteases was added for the quantitative analysis. Refolding of the proteases was achieved by washing the acrylamide gels with a non-ionic detergent (2,5% Triton X-100, 1h). Next, the gels were incubated in an incubation buffer for the digestion of the copolymerized substrate. Finally, the gels were stained with Coomassie Blue and then de-stained until white transparent bands appeared on the blue background. Quantification of the relative levels of individual protein bands was made with the use of internal laboratory standards preparations as detailed elsewhere (23).

### 2.7 Tissue immunofluorescence

Skin wounds were embedded in optimal cutting temperature (OCT) compound, snap frozen in liquid nitrogen and stored at -80°C. Tissue sections 10 µm thick were produced using a cryostat. Sections were fixed with cold methanol for 8 min before blocking with 5% FBS and 1% Fc receptor blocking. Next, skin sections were stained with primary antibody anti-rabbit Cytokeratin 14 (CK14 – Proteintech, USA) and anti-chicken Vimentin (Biolegend) overnight at 4°C. After washing with PBS, the samples were incubated with secondary antibody Alexa Fluor 488-donkey anti-rabbit IgG and Cy3-donkey anti-chicken IgG (Jackson Immunological Labs, UK) for 2 h at room temperature and followed by DNA staining with Hoechst for 30 min. Slides were mounted with ProLong Diamond Antifade Mountant (Thermo Fisher Scientific, USA) and images were acquired on an Andor Dragonfly spinning disk confocal microscope. Quantitative parameters were defined to analyze the images: the wound size measured as the Hoechst-positive plug on top of the lesions, the length of epithelium gap and the length of epithelium tongues.

### 2.8 Wound size

Digital images of the wounds were captured and the wound sizes were quantified with the software ImageJ. Wound healing analysis was expressed as percent healed in relation to wound diameter on day 0 and expressed as area under the curve (AUC).

### 2.9 Scanning electron microscopy

A 100 μl sample from each hydrogel composition was prepared and then fixed using 4% glutaraldehyde in PBS for 30 min. This was followed by drying in an ethanol series. Subsequently, the samples were attached to aluminum stubs and sputter-coated with a 5 nm thick platinum layer under vacuum. The microstructure was then observed using an XL30 FEG scanning electron microscope (Philips, Panama).

### 2.10 Statistical analysis

Data analysis was done using GraphPad Prism 9.1.2. Data were shown as mean ± SEM. Normality test was applied and all data followed a normal distribution. Therefore, Student´s two-tailed unpaired t-test was used when analyzing two groups; otherwise One-Way ANOVA test was used. Statistical significance was determined at p < 0.05.

## 3 Results

### 3.1 Fibrin hydrogel modulates leukocyte subpopulations in cutaneous skin wounds towards resolution

**Figure 1:**
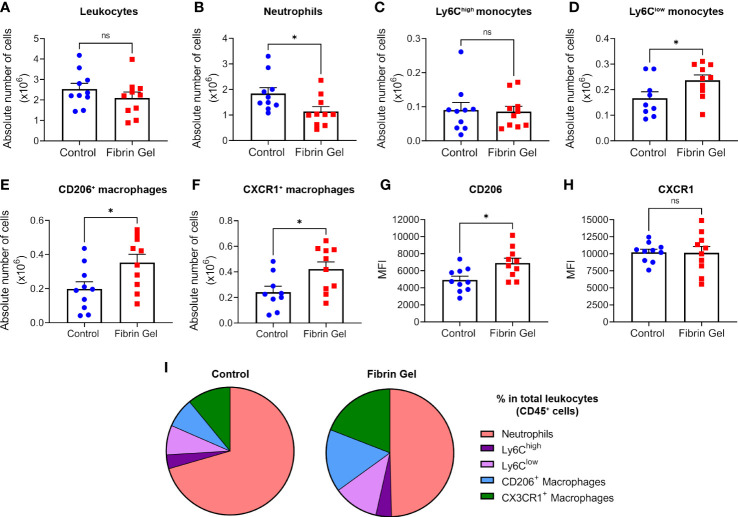
Effect of fibrin hydrogel on leukocyte subpopulations in skin wounds. Leukocyte infiltration was analyzed 3 days postinjury in wounds treated with fibrin hydrogel (Fibrin Gel) and in wounds left untreated (Control). **(A)** Absolute numbers of leukocytes (CD45^+^), **(B)** neutrophils (CD45^+^, CD11b^+^ and Ly6G^+^), **(C)** monocytes Ly6C^high^ (CD45^+^, CD11b^+^, Ly6G^-^, Ly6C^high^), **(D)** monocytes Ly6C^low^ (CD45^+^, CD11b^+^, Ly6G^-^, Ly6C^low^), **(E)** macrophages CD206^+^ (CD45^+^, CD11b^+^, Ly6G^-^, F4/80^+^, CD206^+^) and **(F)** macrophages CX3CR1^+^ (CD45^+^, CD11b^+^, Ly6G^-^, F4/80^+^, CX3CR1^+^). Cell surface expression of CD206 **(G)** and CX3CR1 **(H)** in macrophages population (CD45^+^, CD11b^+^, Ly6G^-^, F4/80^+^) was plotted as Mean Fluorescence Intensity (MFI). **(I)** Comparison of the percentages of leukocyte subpopulations (indicated in color codes) from the total leukocyte population. *p<0.05. Each symbol represents the data from a single mouse. Data are shown as mean ± SEM. ns, not significant

To investigate the role of fibrin in modulating the wound environment, we produced fibrin hydrogel preparations that mimic the natural composition of fibrin clots. Fibrinogen at a similar concentration found in human plasma (3.5 mg/mL) was cleaved by thrombin (0.1 U/mL), generating fibrin monomers that were cross-linked by Factor XIII (0.1 U/mL) for the conversion into fibrin. As indicated in a previous study from our group, this generates a hydrogel with specific structural parameters: roughness average (Ra = 8.1+/- 1.6), fiber diameters of 146.6 +/- 1.1 nm, fiber lengths of 1136,7 +/- 50.2 nm and elastic modules of 752+/- 13 Pa (21). Trace amounts of fibronectin were present in the fibrinogen used and hydrogel stiffness increased in relation with the fibrinogen concentrations (**Supplementary Figures 1A, B**). The physiological fibrin hydrogel (3.5 mg/mL) was added to the wound bed immediately after injury and different parameters of wound repair were analyzed at day 3 post-wound. Leukocyte numbers remained similar in hydrogel-treated wounds compared to control wounds (**Figure 1A**), however, neutrophil counts were lower in the fibrin hydrogel group (**Figure 1B**). Interestingly, when analyzing the monocyte/macrophage infiltration, we observed that, although no difference was seen in the “classical” monocytes Ly6C^high^ population, fibrin hydrogel-treated wounds had higher numbers of Ly6C^low^ monocytes (**Figures 1C, D**). Consistent with these data, higher numbers of macrophages with the capacity to resolve inflammation (CD206^+^ Macrophages and CX3CR1^+^ Macrophages) were observed in wounds treated with fibrin hydrogel (**Figures 1E, F**). The surface expression levels (measured as mean fluorescence intensity, MFI) of the M2 marker CD206 in the total macrophage population were higher in the fibrin hydrogel group, whereas no difference was seen in the expression of CX3CR1 (**Figures 1G, H**). Visualizations of leukocyte subpopulations highlighted the most prominent changes induced by fibrin hydrogel within the leukocyte populations, which included reductions in the percentage of neutrophils and increases of CD206^+^ and CX3CR1^+^ macrophages (**Figure 1I**). In summary, fibrin hydrogel modulated leukocyte subpopulations in skin wounds towards a more resolutive phenotype.

### 3.2 Fibrin hydrogel alters inflammatory molecule profile in cutaneous skin wounds

**Figure 2:**
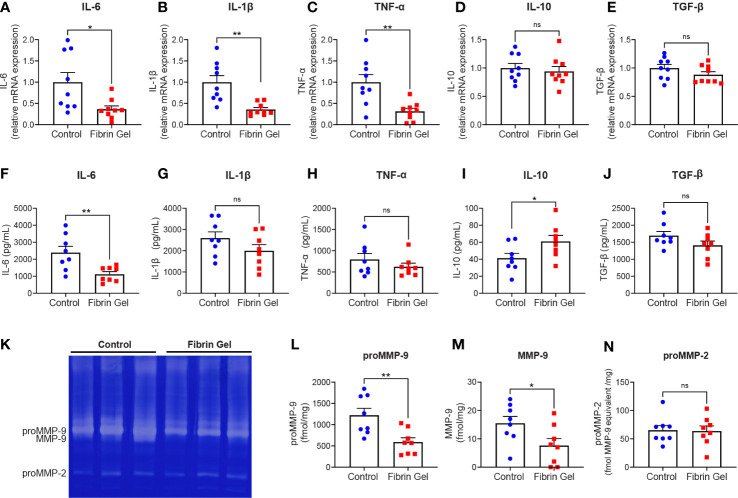
Effect of fibrin hydrogel on cytokine and gelatinase expression in skin wounds. Cytokines and gelatinases were analyzed 3 days postinjury in wounds treated with fibrin hydrogel (Fibrin Gel) and in wounds left untreated (Control). mRNA expression of IL-6 **(A)**, IL1-β **(B)**, TNF-α **(C)**, IL-10 **(D)** and TGF-β **(E)** were determined by qPCR. Concentrations of IL-6 **(F)**, IL-1β **(G)**, TNF-α **(H)**, IL-10 **(I)** and TGF-β **(J)** were determined in wound homogenate by ELISA. Gelatin zymography analysis **(K)** and quantification of gelatinase proteoform expression was done by scanning analysis of pro-MMP-9 **(L)**, activated MMP-9 **(M)** and proMMP-2 **(N)**. *p<0.05, **p<0.01. Each symbol represents the data from a single mouse. Data are shown as mean ± SEM. ns, not significant

Considering the changes observed in leukocyte types induced by fibrin hydrogel, we next investigated profiles of inflammatory molecules in the wounds. Different cytokines were analyzed at mRNA expression and protein secretion/production levels. In agreement with the changes observed in the leukocyte subpopulations, the mRNA expression of pro-inflammatory cytokines IL-6, IL-1β and TNF-α were reduced in fibrin hydrogel-treated wounds compared to control wounds (**Figures 2A–C**). The expression of IL-10 and TGF-β mRNAs did not differ significantly between the two groups (**Figures 2D, E**). At protein secretion/production levels, the amounts of IL-6 were significantly reduced in fibrin hydrogel-treated wounds (**Figure 2F**), while no alterations in the levels of the other pro-inflammatory cytokines IL-1β and TNF-α were observed between the two groups (**Figures 2G, H**). In contrast, increased levels of the anti-inflammatory cytokine IL-10 were seen in fibrin hydrogel-treated wounds (**Figure 2I**) and no differences between groups were seen in the levels of TGF-β (**Figure 2J**).

By gelatin zymography analysis of skin wound tissues, we were able to distinguish between the levels of the two gelatinases MMP-9 and MMP-2. MMP-9 is mainly expressed by neutrophils at sites of acute and excessive inflammation (24, 25). Our analysis revealed that MMP-9 was the most abundant gelatinase found in skin at 3 days after injury, whereas MMP-2 was present at lower levels (**Figure 2K**). The expression of MMP-9 (pro-enzyme and activated forms) was significantly lower in fibrin hydrogel-treated wounds compared to control wounds (**Figures 2L, M**). In contrast, MMP-2 was constitutively expressed within both groups (**Figure 2N**).

Together these data indicated that tailored fibrin hydrogel treatment might diminish the inflammatory response and/or contribute to a faster transition into the resolution phase of inflammation in skin wounds.

### 3.3 Tissue cell population changes by fibrin hydrogel in mouse skin wounds

**Figure 3:**
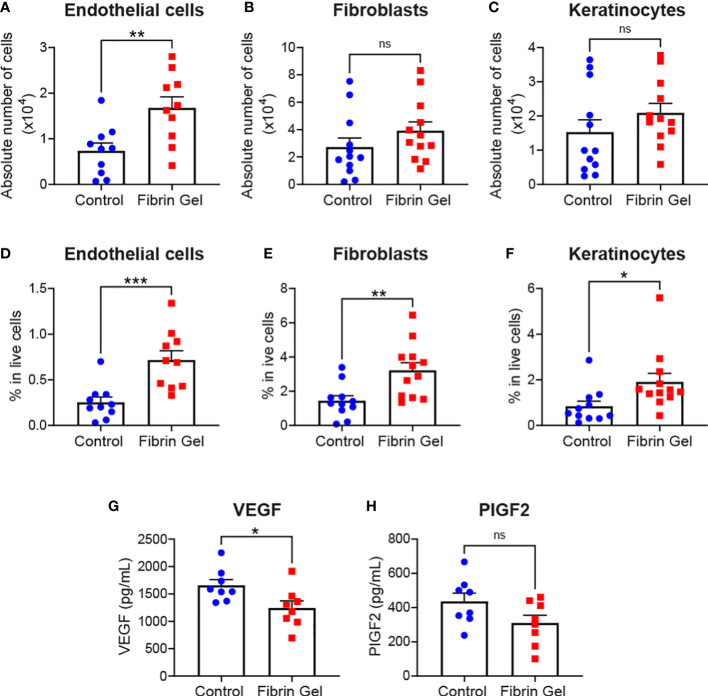
Effect of fibrin hydrogel on stromal cells, keratinocytes and endothelial growth factors in skin wounds. Cell populations and growth factors were analyzed 3 days postinjury in wounds treated with fibrin hydrogel (Fibrin Gel) and in wounds left untreated (Control). **(A)** Absolute numbers of endothelial cells (CD45^-^, CD31^+^), **(B)** fibroblasts (CD45^-^, CD31^-^, CD140a^+^, CD90.2^+^) and **(C)** keratinocytes (CD45^-^, CD31^-^, CD140a^-^, CD49f^+^). Percentages in total live cells were calculated for **(D)** endothelial cells, **(E)** fibroblasts and **(F)** keratinocytes. Concentrations of VEGF **(G)** and PIGF2 **(H)** were determined in wound homogenates by ELISA. *p<0.05, **p<0.01, ***p<0.001. Each symbol represents the data from a single mouse. Data are shown as mean ± SEM. ns, not significant

Aside leukocytes and inflammatory profiles, we also explored the interference of fibrin hydrogel treatment on other cell types essential for effective wound healing. Those included endothelial cells, fibroblasts and keratinocytes. Absolute endothelial cell numbers increased significantly by the fibrin hydrogel treatment, whereas changes in fibroblast and keratinocyte numbers did not reach statistical significance (**Figures 3A–C**). However, when we analyzed the percentages of these three cell populations (relative to total live cells counts), we observed significant increases in the fibrin hydrogel group compared to the control group for the three analyzed cell populations (**Figures 3D–F**). Consequently, the local production of two relevant endothelial cell growth factors, VEGF and PlGF-2, was assessed. Surprisingly, VEGF levels were reduced in the fibrin hydrogel group (**Figure 3G**), whereas no differences between the groups were seen in the levels of PlGF-2 (**Figure 3H**).

**Figure 4:**
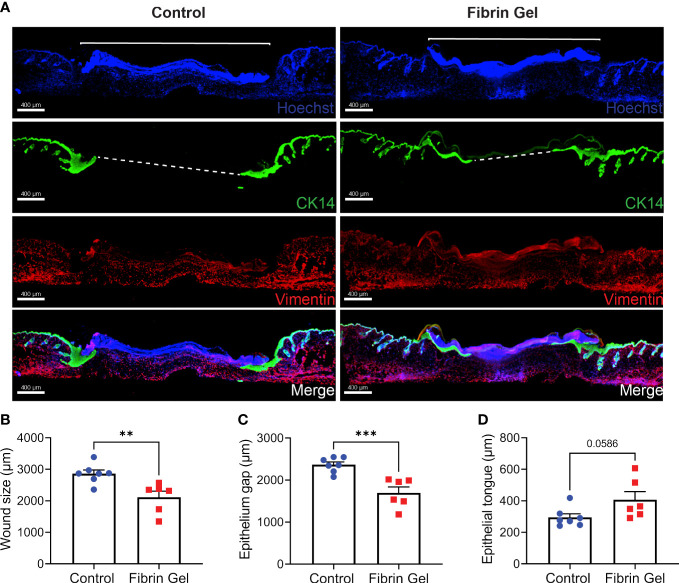
Migration of keratinocytes in wounds treated with fibrin hydrogel. **(A)** Immunofluorescence images of skin wound tissues harvested 3 days postinjury from wounds treated with fibrin hydrogel (Fibrin Gel) and in wounds left untreated (Control). Keratinocytes were stained with cytokeratin 14 (CK14, in green), DNA stained with Hoescht (in blue) and Vimentin in red. Immunofluorescence data analyzed by **(B)** wound size (bracket), **(C)** epithelium gap (striped line) and **(D)** epithelium tongue. **p<0.01, ***p<0.001. Each symbol represents the data from a single mouse. Scale bar: 400 µm. Data are shown as mean ± SEM.

Immunofluorescence analysis of skin wound tissues revealed additional changes induced by fibrin hydrogel. We particularly evaluated the migration of keratinocytes with the use of cytokeratin 14 detection together with Hoechst, that recognized cellular DNA. We also added vimentin staining to provide better overviews of the tissue structures. Keratinocytes migrated below the Hoechst-positive plugs to gradually and in a concentric way reclose the epithelial linings. This phenomenon was visualized as tongue-like structures of keratinocytes growing underneath the plug (**Figure 4A**). Three physical parameters were defined to measure the changes: wound size as the Hoechst-positive plug on top of the lesions, the epithelium gap and the length of epithelial tongues. Fibrin hydrogel treatment resulted in smaller wound sizes and enhanced keratinocyte migrations, evidenced by smaller epithelium gaps and increased epithelial tongue lengths (**Figures 4B–D**).

### 3.4 Fibrin hydrogel accelerates skin wound healing

**Figure 5:**
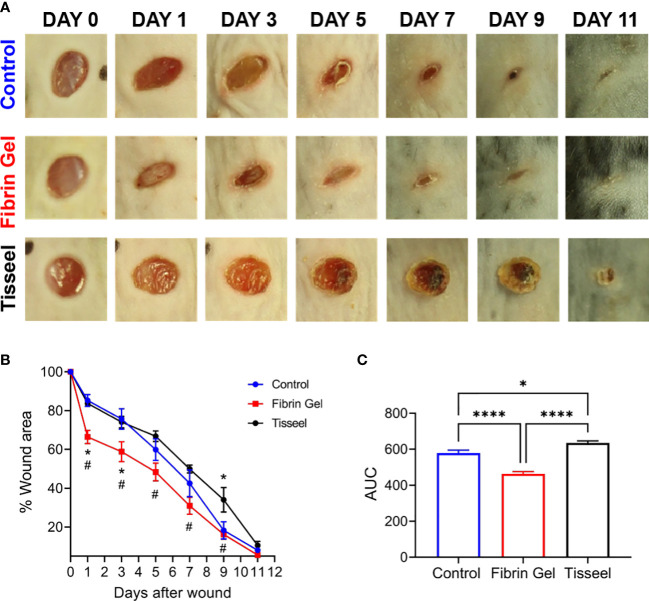
Effect of fibrin hydrogel and Tisseel on mouse skin wound healing. Repair of wounds treated with fibrin hydrogel (Fibrin Gel), fibrin sealant Tisseel or left untreated (Control). **(A)** Representative digital images of wounds up to 11 days after wounding. **(B)** Wound healing expressed as percentage of wound area compared to day 0. n≥5 mice per group, *p<0.05 *vs* Control group, #p<0.05 Fibrin Gel *vs* Tisseel group. **(C)** Wound healing expressed as area under the curve. *p<0.05, ****p<0.0001. Data are shown as mean ± SEM.

**Figure 6:**
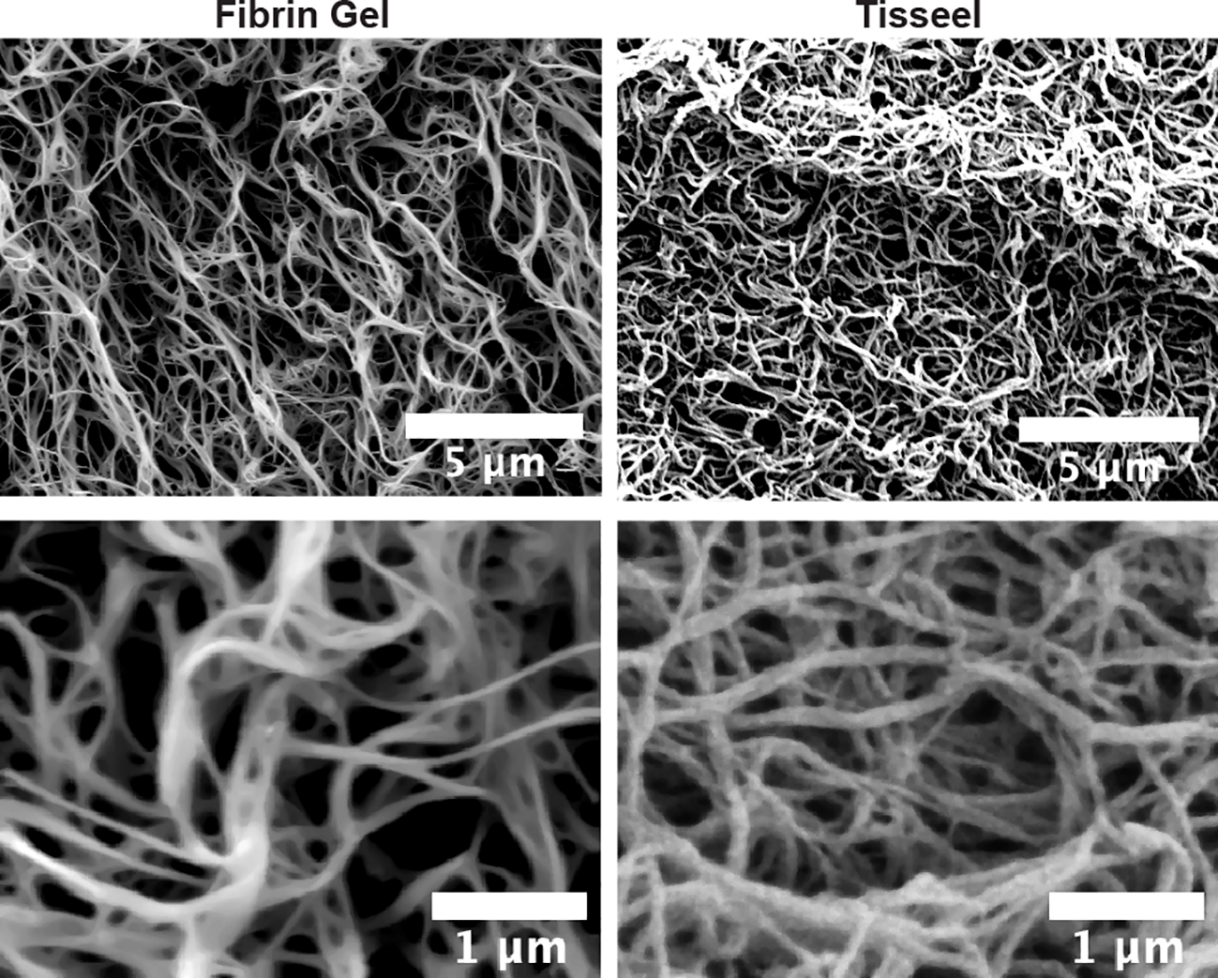
Microstructural differences between fibrin hydrogel and fibrin sealant Tisseel. Scanning electron microscopy (SEM) images of fibrin hydrogel (Fibrin Gel) and Tisseel at low and higher resolution. White bars represent scale indications (5 µm and 1 µm).

As observed above, the positive effects of fibrin hydrogel on key cellular players of the healing process and the alterations in cytokine levels prompted us to further analyze the effect of fibrin hydrogel in wound repair. The evolution of wound sizes was followed until complete recovery, and we compared the effect of the fibrin hydrogel described in our study with that of the commercially available fibrin sealant Tisseel, commonly used for surgical purposes (**Figure 5A**). In contrast to our nearly physiological fibrin preparation, Tisseel is prepared by mixing a rather highly concentrated fibrinogen (72-110 mg/mL), thrombin (500 U/mL) and factor XIII (around 10 U/mL). A significant reduction in wound size was observed in fibrin hydrogel-treated wounds compared to control and Tisseel wounds, at 1 and 3 days post-wound (**Figure 5B**). The statistical difference between fibrin hydrogel and Tisseel-treated wounds was maintained until later time points of the healing process (up to Day 9). In contrast to fibrin hydrogel, Tisseel showed impairment in wound closure compared to control wounds, reaching a significant difference at Day 9. By analyzing the wound area over time, calculated by the area under the curve (AUC), we demonstrated the beneficial outcome of fibrin hydrogel for wound closure and repair and, in contrast, the detrimental outcome of Tisseel (**Figure 5C**). Differences between the two preparations were also observed at the microstructure levels of the fibrin networks. Tisseel showed higher densities of fibrin fibers when compared to our fibrin hydrogel preparation (**Figure 6**). Structural and mechanical characterizations of fibrin microstructures from our hydrogel preparation were previously detailed (21).

## 4 Discussion

Non-healing skin wounds, acute and chronic, are a burden for individual patients and healthcare systems. Vågesjö and colleagues outlined present therapeutic modalities, their limitations (26) and the need for more and better studies. With the discovery of specific growth factors and cytokines, the hope increased to use these in therapeutic settings to improve the granulation of wounds, infiltration of leukocytes for host defense, and wound closure. Regenerative medicine approaches are also being tested with specific progenitor cell types. However, most of these studies have failed so far in reaching the clinic and, for these reasons, we suggested to invest more efforts towards (i) better standardized preclinical animal models, (ii) broad multiparametric analysis of analytes and (iii) development of pharmacological approaches aiming to modulate endogenous cells and molecules within the host (2). These elements are here illustrated in an animal model of skin wound healing.

We prepared a tailored fibrin scaffold that mimics the natural composition of a fibrin clot, in consideration of the physiological concentration of fibrinogen found in human plasma. We showed that fibrin hydrogel induced a more resolutive phenotype in wound macrophages and shifted the pro-inflammatory cytokines profiles within the wounds towards restorative profiles. Moreover, fibrin hydrogel increased local tissue cells, such as endothelial cells, fibroblasts and keratinocytes and enhanced keratinocyte migration. Lastly, we showed that, differently from the commercially available fibrin sealant Tisseel, the formulated fibrin hydrogel accelerated the healing of mouse skin wounds.

The importance of fibrin(ogen) for an adequate skin wound repair was evident in fibrinogen-deficient mice, in which an abnormal pattern of tissue repair was observed, including altered cell migration and instability of granulation tissue (27). The need of this provisional matrix to support the progression of tissue repair raises fibrin to the level of a pharmacological agent with possible applications and therapeutic benefits. Although the addition of fibrin to various matrices into wound beds has been used in other studies, limited information exists about the single contribution of fibrin as a therapeutic approach for wound healing (28–30). In most studies, combinations of fibrin with other proteins, platelets, cells and/or growth factors were used and, therefore, these studies fall short in defining the beneficial effect of fibrin itself (20, 31–33). For instance, in a novel fibrin-fibronectin matrix, the beneficial effect in wound healing was attributed to a high fibronectin content (31). In contrast, our fibrin preparation contains low amounts of fibronectin. Nevertheless, in accordance with Jara et al. (31), our study also highlights the importance of application of physiological concentrations of fibrinogen when using fibrin matrix for wound repair applications.

Fibrin interacts with several integrin and nonintegrin receptors on multiple cell types. In leukocytes, fibrin(ogen) binds to αMβ2 (CD11b/CD18, Mac-1) integrins and mediates inflammatory leukocyte functions (34). Interestingly, this interaction is conformation-dependent, being strong when fibrinogen is immobilized or converted to fibrin, but weak with soluble fibrinogen (35). In our study, populations and phenotypes of leukocytes were altered by fibrin hydrogel treatment, suggesting fibrin-cell interactions. The increase in the Ly6C^low^ monocytes/macrophages population in fibrin hydrogel-treated wounds indicated a higher influx of this non-classical Ly6C^low^ phenotype induced by fibrin. In skin wounds, a distinct phenotype was previously described for monocyte populations, in which Ly6C^high^ and Ly6C^low^ populations display an inflammatory and anti-inflammatory profile, respectively (36). The transition of cell phenotype is also well known for macrophage populations. The shift from pro-inflammatory (M1-like phenotypes) to anti-inflammatory (M2-like phenotypes) is linked with the resolution of inflammation and represents a critical step for wound repair (9). In the present work, increased numbers of resolutive macrophages expressing CX3CR1 or CD206 receptors were seen in wounds treated with fibrin hydrogel as well as increased expression of CD206. Multiple reparative processes in wound healing have been attributed to CX3CR1 expression in macrophages and are linked with restorative profiles (37, 38). Similarly, CD206 is upregulated in anti-inflammatory macrophages and commonly used as an M2 marker (39). The change in macrophage phenotype from pro-inflammatory to anti-inflammatory is often impaired in chronic and non-healing wounds, which leads to a persisting inflammatory response (40–42). Therefore, a wide range of approaches towards promoting anti-inflammatory macrophages has been investigated, including the use of biomaterials and scaffolds providing microenvironmental cues to modulate endogenous macrophages (9, 14, 15). In agreement with our findings, *in vitro* studies have shown the interference of fibrin in modulating macrophages towards a more resolutive phenotype (43, 44). Accordingly, Tanaka et al. showed that fibrin hydrogel implanted subcutaneously could promote the recruitment of anti-inflammatory macrophages, suggesting fibrin as an optimal biomaterial for macrophage-induced regenerative therapies (44). Pro-resolving macrophages suppress inflammation by downregulating and upregulating inflammatory and anti-inflammatory mediators, respectively. In agreement with the resolutive phenotype induced by fibrin hydrogel on wound macrophages, decreased inflammatory profiles were also observed in the present study, including downregulation of pro-inflammatory cytokines and MMP-9, and upregulation of the anti-inflammatory cytokine IL-10. Higher expression of MMP-9 protein is commonly associated with impaired chronic wounds once it is correlated with a higher and uncontrolled inflammatory stage in these conditions (45).

Additional support for therapeutic effects is given here by the increase in endothelial cell, fibroblast and keratinocyte populations. The positive effect of fibrin hydrogel on these cell populations in the skin might represent an indirect effect as a result of the interference of fibrin in the previous phase of inflammation or a direct effect of fibrin on these three cell populations. Fibrin provides a permissive matrix for angiogenesis and endothelial cells directly interact with fibrin *via* integrin receptors and VE-Cadherin (46–48). In addition, several growth factors bind to fibrin, including VEGF, a major player in angiogenesis (49). Surprisingly, VEGF levels were reduced in wounds treated with fibrin hydrogel. The absence of elevated levels of VEGF in the fibrin hydrogel group might be explained by the presence of other players involved in the migration of the endothelial cells (50). Additionally, fibrin-derived E-fragment has been shown to possess angiogenic activity on its own and to enhance the angiogenic effects of VEGF (51, 52). Future studies would be necessary to unravel the underlying mechanism. Similar to endothelial cells, fibroblasts also directly interact with fibrin through integrin receptor αvβ3 (53). Fibroblasts proliferate and migrate into fibrin matrices and are responsible for remodeling the fibrin matrix into a collagen-rich matrix (54–56). Fibrin also promotes keratinocyte migration *in vitro* through a plasminogen-mediated migration (57, 58). In agreement with this, our fibrin hydrogel preparation enhanced keratinocyte migration *in vivo*.

Aside molecular and cellular interactions by fibrin, also biomechanical properties of fibrin hydrogel might play a role. Variations in the concentration of clotting proteins and ion strengths influence the structure of the fibrin network and, consequently, impact on mechanical properties and biological functions of fibrin. Higher fibrinogen concentrations relate to higher stiffness of fibrin matrix and decreased fibroblast proliferation (59). Fibroblast migration was also affected by alterations in fibrinogen concentrations of fibrin gel matrices, 3mg/mL being optimal (55). Similarly, high fibrinogen concentrations were shown to impair the migration of keratinocytes (58). Moreover, high factor XIII concentrations tighten the coupling between the protofibrils, making the fiber network stiffer and less porous (60). Likewise, fibrin sealants with higher thrombin concentration formed tighter fibers, affecting negatively proliferation and viability of keratinocytes and fibroblasts (29, 61). In addition, Gugerell et al. described that high thrombin levels negatively affect *in vivo* rat skin wound healing, although interference of other factors, such as fibrinogen, were not excluded (29). We aimed here to generate a native fibrin network and, therefore, used physiological concentrations of fibrinogen in our preparations. Biomaterial properties were previously detailed for our preparations (21). The stiffness of our hydrogel preparations is similar to values of a blood clot, around 1 kPa (62). These preparations differ significantly from commercial preparations, such as Tisseel, that contains high concentrations of clotting proteins and stiffness around 100 kPa (22, 63). Discrepancies in fiber network structure and in the response of these two fibrin preparations on skin wound healing were described here. Together with our findings, the studies mentioned above highlighted beneficial effects of modifications in fibrin compositions for wound healing application.

The influence of biomaterial properties on cellular behavior and function is not limited to fibrin matrices. Growing interest exists in understanding the influence of different biomaterial properties and scaffold architecture on macrophage polarization (15). For instance, material stiffness alters macrophage polarization, function and migration. Specifically, stiff hydrogels induce a pro-inflammatory phenotype on macrophages, with decreased phagocytic and migration capacities, whereas in softer gels macrophages are primed towards an anti-inflammatory phenotype (64).

Another critical point for wound repair is the degradation of the provisional matrix formed by fibrin to allow the subsequential steps of repair, including granulation tissue formation and tissue remodeling into a collagen-rich matrix. In particular, physiological fibrinolysis is done by the ubiquitous plasminogen, present in plasma and plasma exudates (e.g. in skin wounds). For fibrinolysis to occur, this plasminogen needs to be activated by one of the two major plasminogen activators (constitutive urokinase/u-PA or inducible tissue-type plasminogen activator/t-PA) (65). The importance of fibrin matrix degradation for wound repair is clearly shown in plasminogen-deficient mice (66, 67). Therefore, the use of biomaterials that can be physiologically degraded is an important point to consider. The architecture of the fibrin network determines the fibrinolysis rate and consequently, a tight fibrin network, containing high thrombin and fibrinogen concentrations, is degraded at slower rate than loose ones (29, 68, 69). In the present study, we used conditions to obtain physiological fibrin hydrogels and therefore, we expect the hydrogels degradation to be similar to a normal fibrinolysis process. In contrast, a regular fibrinolysis might be insufficient to complete the timely degradation of Tisseel, which contains high concentrations of clotting proteins, possess higher fiber density and contains aprotinin, a plasmin inhibitor that prevents fibrinolysis. Therefore, the detrimental effect of Tisseel in wound healing could also be associated with its higher fiber density and slower fibrinolysis rate. At earlier moments in the healing process, dense fibrin networks might act as a barrier, impairing cell migration. At later moments, high fiber density can slow the fibrinolysis rate and subsequently interfere with the following steps of wound healing. Fibrin deposition due to plasminogen deficiency is linked with a sustained and chronic inflammation (70, 71).

A persistent inflammatory response is often a significant challenge in the management of chronic wounds, such as in diabetic patients. Based on the immunomodulatory effect of fibrin hydrogel demonstrated in this study, we believe that the application of fibrin hydrogels to chronic wounds holds great promise. Thus, further investigation into this potential therapeutic approach is valuable and may lead to improved outcomes for patients with chronic wounds. In infected wounds however, this therapeutic approach might be hampered by staphylokinase and streptokinase, potent fibrinolytic proteins produced by Staphylococcus and Streptococcus species, respectively (72).

A limitation of our study is wound contraction *versus* re-epithelialization. Whereas our model is commonly used in wound healing and skin regeneration research, it comes with disadvantages in rodents and other animals. Unlike in humans, in rodents skin contraction is a mechanism that contributes to wound closure (73). This implies that the collection of tissues for the analysis of cells may be confounded by such contraction. Another limitation of our study is related to the fact that wound healing is a highly dynamic process. We recognized that sampling at day 3 provides only one (important) time frame of the process. As specific mRNA production and breakdown precedes or coincides with protein synthesis and secretion, we did not observe full concordance between the measured mRNA and protein levels.

In conclusion, we described a tailored fibrin hydrogel which induces considerable changes in the skin wound environment towards a faster repair. These beneficial effects of fibrin hydrogel might be attributed to biochemical features of fibrin acting as a cell adhesion ligand, together with biomechanical and biophysical cues of the formulated fibrin hydrogel structure. Our work incites similar clinical studies in humans.

## Data availability statement

The original contributions presented in the study are included in the article/**Supplementary Material**. Further inquiries can be directed to the corresponding author.

## Ethics statement

The animal study was reviewed and approved by Animal Ethics Committee of KU Leuven (License numbers P270/2015, P128/2019).

## Author contributions

Conceptualization and supervision: RP, ME, GO. Data curation and analysis: RP, ME, EU-B, EM, BM-D, JV. Funding acquisition: JJV, GO. First draft preparation: RP, GO. All authors contributed to the article and approved the submitted version.
