# Supplementary material for: Physiological fibrin hydrogel modulates immune cells and molecules and accelerates mouse skin wound healing
Source: Front Immunol. 2023 Apr 24;14:1170153. doi: 10.3389/fimmu.2023.1170153 (PMC10165074; doi:10.3389/fimmu.2023.1170153)
Supplement: Supplementary file 1 [file DataSheet_1.docx]

Supplementary Material

Physiological fibrin hydrogel modulates immune cells and molecules and accelerates mouse skin wound healing

Rafaela Vaz Sousa Pereira^1^, Mostafa EzEldeen^2,3^, Estefania Ugarte-Berzal^1^, Erik Martens^1^, Bert Malengier-Devlies^1^, Jennifer Vandooren^1^, Jan Jeroen Vranckx^4^, Patrick Matthys^1^, Ghislain Opdenakker^1*^

*** Correspondence:** Ghislain Opdenakker, ghislain.opdenakker@kuleuven.be

**
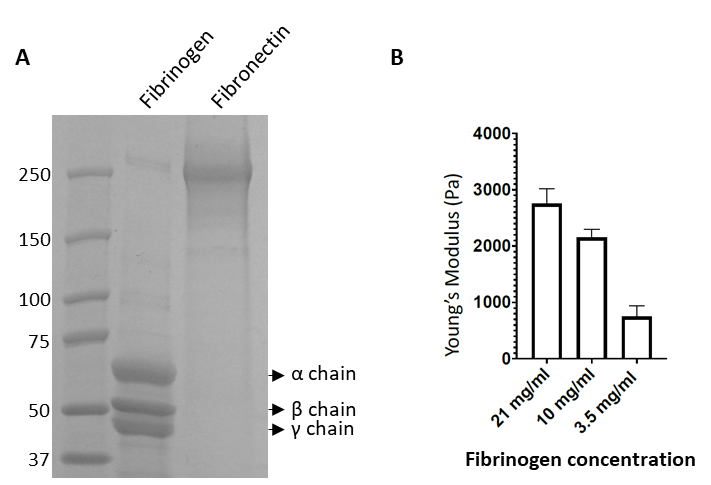
**

**Supplementary Figure 1: Purity of fibrinogen and elastic modulus of Fibrin hydrogel preparations.** (**A**) Preparations of 10 µg human fibrinogen or recombinant fibronectin were separated by SDS-Polyacrylamide gel electrophoresis and stained with Coomassie Brilliant Blue. A standard mixture of proteins with known molecular weight was included in the left lane. (**B**) Fibrin hydrogels were prepared with different concentrations of fibrinogen (3.5 mg/mL, 10 mg/mL and 21 mg/mL) and the stiffness was determined as previously described (1) and expressed as Young’s Modulus in Pascal (Pa).

#
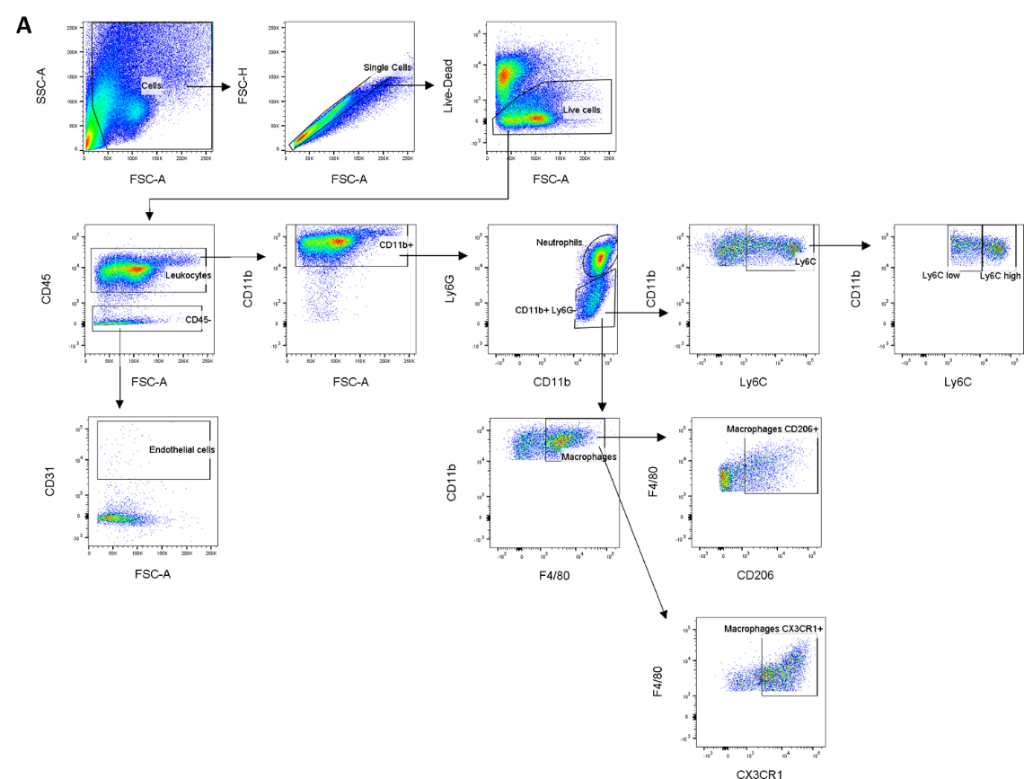


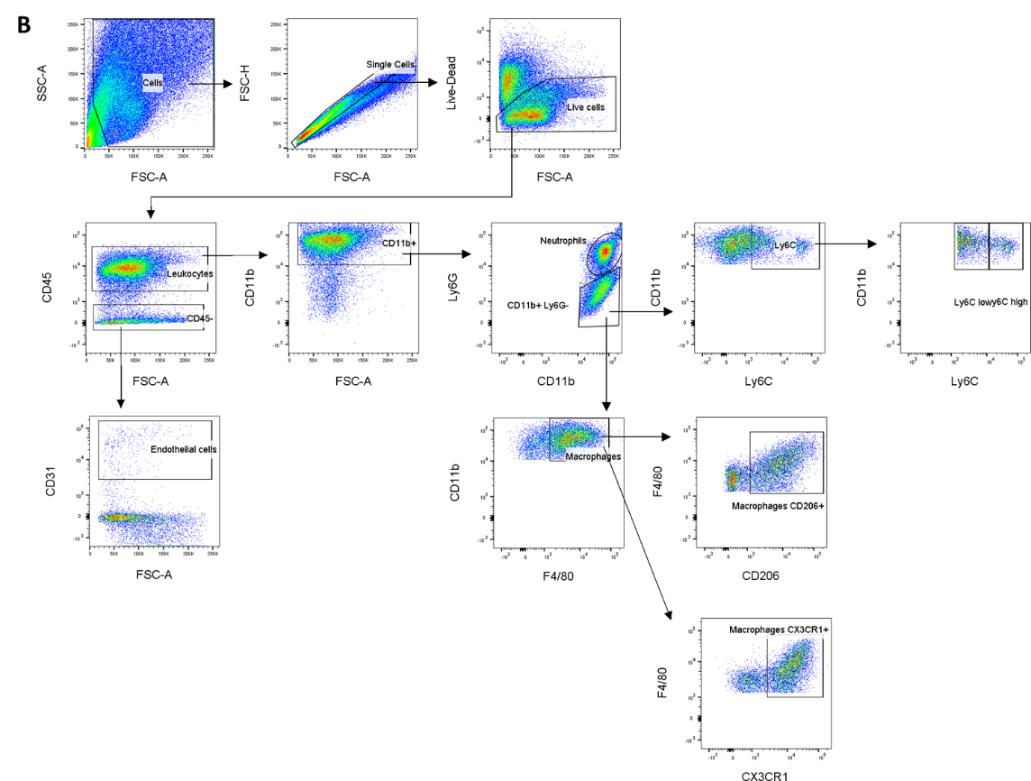


**Supplementary Figure 2: Gating strategy used for flow cytometry analysis of leukocytes (myeloid cells) and endothelial cells in mouse skin wounds.** At Day 3 after wounding, skin cells were isolated and stained for flow cytometry. Gating strategy used in (**A**) control wounds and (**B**) fibrin hydrogel-treated wounds. After selection of single and live cells, the interest cells were defined as follows: leukocytes (CD45^+^), neutrophils (CD45^+^, CD11b^+^ and Ly6G^+^), monocytes Ly6C^high^ (CD45^+^, CD11b^+^, Ly6G^-^, Ly6C^high^), monocytes Ly6C^low^ (CD45^+^, CD11b^+^, Ly6G^-^, Ly6C^low^), macrophages CD206^+^ (CD45^+^, CD11b^+^, Ly6G^-^, F4/80^+^, CD206^+^), macrophages CX3CR1^+^ (CD45^+^, CD11b^+^, Ly6G^-^, F4/80^+^, CX3CR1^+^) and endothelial cells (CD45^-^, CD31^+^).


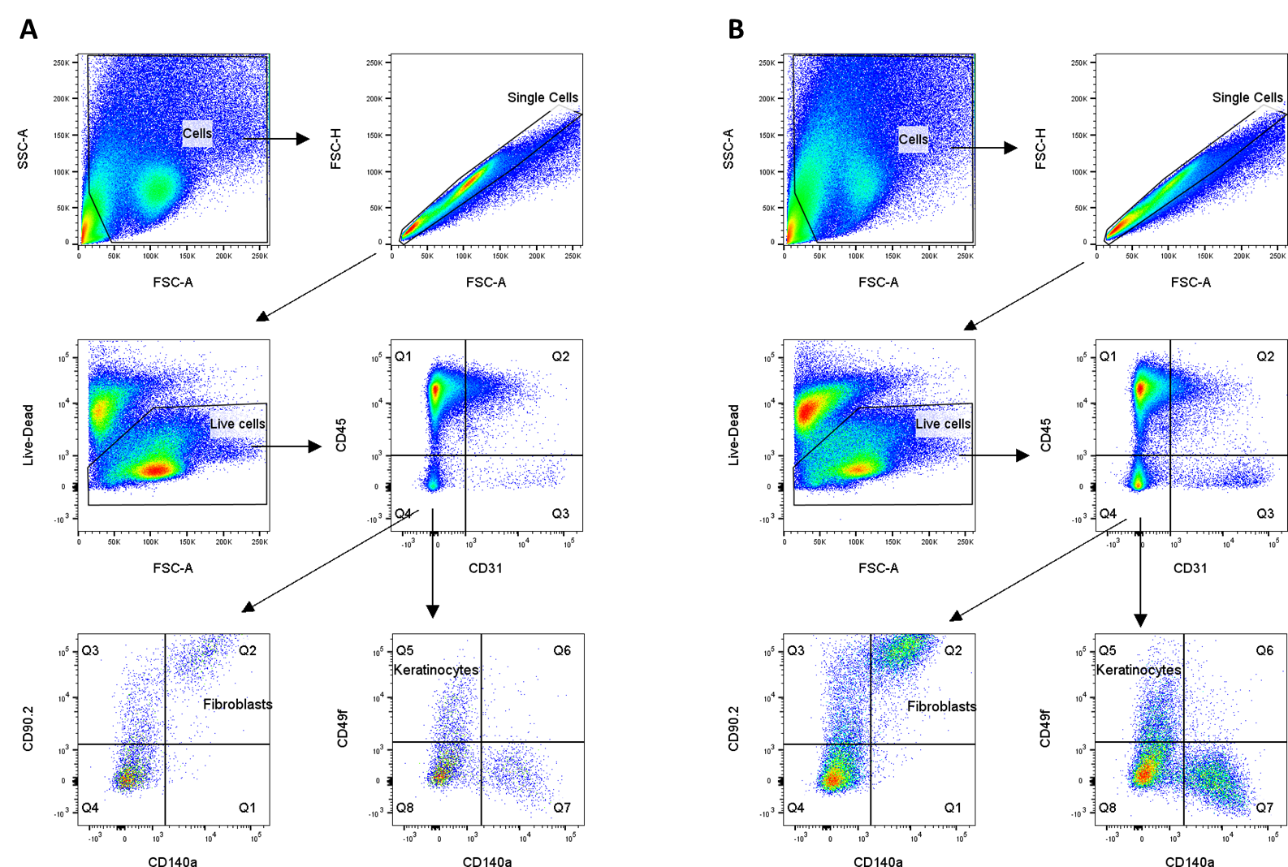


**Supplementary Figure 3: Gating strategy used for flow cytometry analysis of fibroblasts and keratinocytes in mouse skin wounds.** At Day 3 after wounding, skin cells were isolated and stained for flow cytometry. Gating strategy used in (**A**) control wounds and (**B**) fibrin hydrogel-treated wounds. After selecting single and live cells, fibroblasts were defined as CD45^-^, CD31^-^, CD140a^+^ and CD90.2^+^ cells and keratinocytes were defined as CD45^-^, CD31^-^, CD140a^-^ and CD49f^+^ cells.

**Supplementary Table 1. Antibodies used for Flow cytometry analysis**

| **Panel 1: Leukocytes (myeloid cells) and endothelial cells** | | | | |
| --- | --- | --- | --- | --- |
| **Antibody** | **Clone** | **Fluorophore** | **Company** | **Cell specificities** |
| CD45 | 30-F11 | BUV395 | BD | Leukocytes |
| CD11b | M1/70 | BV786 | BD | Myeloid cells |
| Ly6G | 1A8 | BV421 | eBioscience | Neutrophils |
| Ly6C | HK1.4 | PerCP-Cy5.5 | eBioscience | Monocytes |
| F4/80 | BM8 | PE/Cy5 | eBioscience | Macrophages |
| CD206 | C068C2 | PE | Biolegend | Macrophages |
| CX3CR1 | SA011F11 | APC | Biolegend | Macrophages |
| CD31 | MEC 13.3 | APCR700 | BD | Endothelial cells |
| **Panel 2: Keratinocytes and Fibroblasts** | | | |  |
| **Antibody** | **Clone** | **Fluorophore** | **Company** | **Cell specificities** |
| CD45 | 30-F11 | BUV395 | BD | Leukocytes |
| CD140a | APA5 | BV421 | Biolegend | Fibroblasts |
| CD90.2 | 30-H12 | BV785 | Biolegend | Fibroblasts |
| CD49f | GoH3 | APC | Biolegend | Keratinocytes |
| CD31 | MEC 13.3 | APCR700 | BD | Endothelial cells |

**Supplementary Table 2. Primers used for qPCR analysis**

| **Protein** | **Gene** | **Dye** | **Exon Location** | **Catalog number and Company** |
| --- | --- | --- | --- | --- |
| Interleukin 6 (IL-6) | Il6 | FAM | 4-5 | Mm.PT.58.10005566 |
| Tumor Necrosis Factor alpha (TNF-α) | Tnf | FAM | 2-4 | Mm.PT.58.12575861 |
| Interleukin 1 beta (IL-1β) | Il1b | FAM | 1-3 | Mm.PT.58.42940223 |
| Interleukin 10 (IL-10) | Il10 | FAM | 3-5 | Mm.PT.58.13531087 |
| Transforming Growth Factor 1 (TGF-β) | Tgfb | FAM | 1-2 | Mm.PT.58.11254750 |
| 18S ribosomal RNA (18S) | RNA18S5 | FAM | 1-1 | Hs.PT.39a.22214856.g |

**Reference:**

1. EzEldeen M, Toprakhisar B, Murgia D, Smisdom N, Deschaume O, Bartic C, et al. Chlorite oxidized oxyamylose differentially influences the microstructure of fibrin and self assembling peptide hydrogels as well as dental pulp stem cell behavior. Sci Rep. 2021;11(1):5687.
